# The complete genome sequences of sulfur-oxidizing *Gammaproteobacteria Sulfurifustis variabilis* skN76^T^ and *Sulfuricaulis limicola* HA5^T^

**DOI:** 10.1186/s40793-016-0196-0

**Published:** 2016-09-15

**Authors:** Kazuhiro Umezawa, Tomohiro Watanabe, Aya Miura, Hisaya Kojima, Manabu Fukui

**Affiliations:** The Institute of Low Temperature Science, Hokkaido University, Kita-19, Nishi-8, Kita-ku, Sapporo, 060-0819 Japan

**Keywords:** Bacteria, Gram-negative, Sulfur-oxidizing bacteria, *Acidiferrobacterales*, *Acidiferrobacteraceae*

## Abstract

*Sulfurifustis variabilis* and *Sulfuricaulis limicola* are autotrophic sulfur-oxidizing bacteria belonging to the family *Acidiferrobacteraceae* in the order *Acidiferrobacterales*. The type strains of these species, strain skN76^T^ and strain HA5^T^, were isolated from lakes in Japan. Here we describe the complete genome sequences of *Sulfurifustis variabilis* skN76^T^ and *Sulfuricaulis limicola* HA5^T^. The genome of *Sulfurifustis variabilis* skN76^T^ consists of one circular chromosome with size of 4.0 Mbp including 3864 protein-coding sequences. The genome of *Sulfuricaulis limicola* HA5^T^ is 2.9 Mbp chromosome with 2763 protein-coding sequences. In both genomes, 46 transfer RNA-coding genes and one ribosomal RNA operon were identified. In the genomes, redundancies of the genes involved in sulfur oxidation and inorganic carbon fixation pathways were observed. This is the first report to show the complete genome sequences of bacteria belonging to the order *Acidiferrobacterales* in the class *Gammaproteobacteria*.

## Introduction

*Sulfurifustis variabilis* skN76^T^ and *Sulfuricaulis limicola* HA5^T^ are gammaproteobacterial sulfur-oxidizing bacteria isolated from sediments of Lake Mizugaki and Lake Harutori, respectively [1, 2]. They both belong to the family *Acidiferrobacteraceae* in the order *Acidiferrobacterales*. In this order, only three species have been isolated in pure culture. They are all chemolithoautotrophs and can grow by oxidation of inorganic sulfur compounds. *Sulfurifustis variabilis* and *Sulfuricaulis limicola* are neutrophilic, whereas the other species, *Acidiferrobacter thiooxydans*, is acidophilic [3]. Taxonomy of *Acidiferrobacter thiooxydans* has been revised several times, and the family *Acidiferrobacteraceae* and order *Acidiferrobacterales* were recently established to accommodate the species [1, 3–5]. The members of the family *Acidiferrobacteraceae* have been frequently detected in various environments as gene sequences [2, 3, 6].

Here we show the complete genome sequences of *Sulfurifustis variabilis* skN76^T^ and *Sulfuricaulis limicola* HA5^T^ as the first genomes of the order *Acidiferrobacterales*.

## Organism information

### Classification and features

**Fig. 1:**
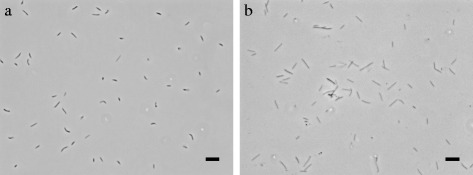
Phase-contrast micrographs of *Sulfurifustis variabilis* skN76^T^ (**a**) and *Sulfuricaulis limicola* HA5^T^ (**b**), grown with thiosulfate at 45 and 28 °C, respectively. Bars, 5 μm

**Fig. 2:**
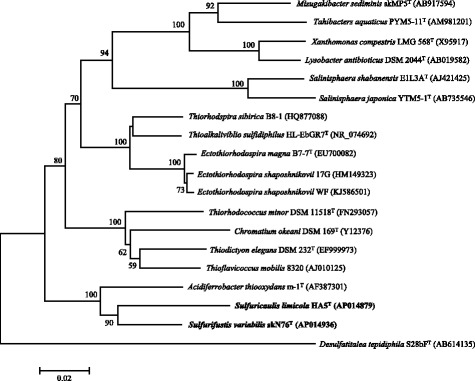
Phylogenetic tree showing the relationships of *Sulfurifustis variabilis* skN76^T^ and *Sulfuricaulis limicola* HA5^T^ with other members of the class *Gammaproteobacteria* based on 16S rRNA gene sequences aligned by using CLUSTAL W. *Desulfatitalea tepidiphila* S28bF^T^ was used as an outgroup. This tree was reconstructed using 1412 sites with the neighbor-joining method by using MEGA6 [27]. Percentage values of 1000 bootstrap resamplings are shown at nodes; values below 50 % were not shown

The cells of *Sulfurifustis variabilis* skN76^T^ are rod-shaped or filamentous form with varying length, and 0.3–0.5 μm in width (Fig. 1a, Table 1). The cells of *Sulfuricaulis limicola* HA5^T^ are rod-shaped, 1.2–6.0 μm in length and 0.3–0.5 μm in width (Fig. 1b, Table 1). They are both Gram-stain-negative. *Sulfurifustis variabilis* and *Sulfuricaulis limicola* belong to the family *Acidiferrobacteraceae* within the class *Gammaproteobacteria* (Fig. 2). They both utilized thiosulfate, tetrathionate and elemental sulfur as electron donors for chemolithoautotrophic growth under aerobic conditions [1, 2].

**Table 1:** Classification and general features of *Sulfurifustis variabilis* skN76^T^ and *Sulfuricaulis limicola* HA5^T^ according to MIGS recommendations

MIGS ID	Property	*Sulfurifustis variabilis* skN76^T^		*Sulfuricaulis limicola* HA5^T^	
		Term	Evidence code ^a^	Term	Evidence code ^a^
	Classification	Domain *Bacteria*	TAS [23]	Domain *Bacteria*	TAS [23]
		Phylum *Proteobacteria*	TAS [24]	Phylum *Proteobacteria*	TAS [24]
		Class *Gammaproteobacteria*	TAS [25]	Class *Gammaproteobacteria*	TAS [25]
		Order *Acidiferrobacterales*	TAS [1]	Order *Acidiferrobacterales*	TAS [1]
		Family *Acidiferrobacteraceae*	TAS [1]	Family *Acidiferrobacteraceae*	TAS [1]
		Genus *Sulfurifustis*	TAS [1]	Genus *Sulfuricaulis*	TAS [2]
		Species *Sulfurifustis variabilis*	TAS [1]	Species *Sulfuricaulis limicola*	TAS [2]
		Type strain skN76		Type strain HA5	
	Gram stain	negative	TAS [1]	negative	TAS [2]
	Cell shape	rod or filaments	TAS [1]	rod	TAS [2]
	Motility	motile	TAS [1]	not reported	
	Sporulation	not reported		not reported	
	Temperature range	28–46 °C	TAS [1]	8–37 °C	TAS [2]
	Optimum temperature	42–45 °C	TAS [1]	28–32 °C	TAS [2]
	pH range; Optimum	6.3–8.9; 6.8–8.2	TAS [1]	6.1–9.2; unknown	TAS [2]
	Carbon source	bicarbonate	TAS [1]	bicarbonate	TAS [2]
MIGS-6	Habitat	Sediment of a lake	TAS [1]	Sediment of a lake	TAS [2]
MIGS-6.3	Salinity	<2.6 % NaCl (w/v)	TAS [1]	<1.2 % NaCl (w/v)	TAS [2]
MIGS-22	Oxygen requirement	aerobic	TAS [1]	aerobic	TAS [2]
MIGS-15	Biotic relationship	free-living	TAS [1]	free-living	TAS [2]
MIGS-14	Pathogenicity	non-pathogen	NAS	non-pathogen	NAS
MIGS-4	Geographic location	Lake Mizugaki, Japan	TAS [1]	Lake Harutori, Japan	TAS [2]
MIGS-5	Sample collection	November 30, 2010	NAS	April 26, 2012	NAS
MIGS-4.1	Latitude	35°51.5′ N	TAS [26]	42°58.4′ N	NAS
MIGS-4.2	Longitude	138°30.0′ E	TAS [26]	144°23.9′ E	NAS
MIGS-4.4	Altitude	not reported		not reported	

^a^ Evidence codes–*IDA* Inferred from Direct Assay, *TAS* Traceable Author Statement (i.e., a direct report exists in the literature), *NAS* Non-traceable Author Statement (i.e., not directly observed for the living, isolated sample, but based on a generally accepted property for the species, or anecdotal evidence). These evidence codes are from the Gene Ontology project

## Genome sequencing information

### Genome project history

*Sulfurifustis**variabillis* skN76^T^ and *Sulfuricaulis limicola* HA5^T^ were selected for sequencing as representatives of sulfur-oxidizing bacteria belonging to the order *Acidiferrobacterales*, to reveal characteristics of their genomes. A summary of the project information is shown in Table 2.

**Table 2:** Project information

MIGS ID	Property	*Sulfurifustis variabilis* skN76^T^	*Sulfuricaulis limicola* HA5^T^
		Term	Term
MIGS 31	Finishing quality	Completed	Completed
MIGS-28	Libraries used	15–20 kb SMRTbell^TM^ library	10–20 kb SMRTbell^TM^ library
MIGS 29	Sequencing platforms	PacBio RS II	PacBio RS II
MIGS 31.2	Fold coverage	210 ×	142 ×
MIGS 30	Assemblers	RS_HGAP Assembly.2	RS_HGAP Assembly.3
MIGS 32	Gene calling method	Microbial Genome Annotation Pipeline	Microbial Genome Annotation Pipeline
	Locus Tag	SVA	SCL
	Genbank ID	AP014936	AP014879
	GenBank Date of Release	July 29, 2016	July 29, 2016
	BIOPROJECT	PRJDB4108	PRJDB3927
MIGS 13	Source Material Identifier	DSM 100313	DSM 100373
	Project relevance	Environmental	Environmental

### Growth conditions and genomic DNA preparation

*Sulfurifustis variabilis* skN76^T^ and *Sulfuricaulis limicola* HA5^T^ were grown with 20 mM thiosulfate as an energy source in a bicarbonate-buffered medium previously described [1], at 45 and 28 °C, respectively. Genomic DNA samples were prepared by using Wizard® genomic DNA purification kit (Promega, Madison, WI, USA) from approximately 0.2 ml (skN76) or 0.1 ml (HA5) of cell pellets. Amounts of the obtained DNA assessed by spectrophotometry were *ca.* 270 μg (skN76) and 90 μg (HA5) respectively, and the UV absorption ratio of 260/280 nm was greater than 1.8 in both samples.

### Genome sequencing and assembly

The genomic DNA was sheared into approximately 20 kb using g-TUBE (Covaris, Inc., Woburn, MA, USA). The SMRTbell^TM^ templates were prepared from the fragments using SMRTbell^TM^ Template Prep Kit 1.0 (Pacific Biosciences, Menlo Park, CA, USA). The size-selected libraries for sequencing were prepared by using BluePippin (Sage Science, Baverly, MA, USA). The libraries were sequenced on a PacBio RS II instrument (Pacific Biosciences) with P6-C4 chemistry (for *Sulfurifustis**variabillis* skN76^T^) or P5-C3 chemistry (for *Sulfuricaulis limicola* HA5^T^). *De novo* assembly was performed by using RS_HGAP Assembly.3 (for *Sulfurifustis**variabillis* skN76^T^) or RS_HGAP Assembly.2 (for *Sulfuricaulis limicola* HA5^T^), implemented within the SMRT Analysis v2.3 (Pacific Biosciences) software environment. By assembling 79,017 subreads (837,333,548 bp) of *Sulfurifustis**variabillis* skN76^T^, two contigs with the lengths of *ca*. 4.0 Mbp and *ca*. 5.4 kbp were obtained. The shorter one was identical to a partial sequence of the larger one, and a circular chromosome was manually constructed from the larger contig by finding self-overlapping regions using the *in silico* Molecular Cloning (R) Genomic Edition (In Silico Biology, Inc., Yokohama, Japan) application. As for *Sulfuricaulis limicola* HA5^T^, a single contig (*ca*. 2.9 Mbp) was obtained by assembling 61,565 subreads (409,124,339 bp), and circular chromosome was manually constructed in the same manner.

### Genome annotation

The genomes were annotated automatically using the Microbial Genome Annotation Pipeline [7]. Further manual annotation of the predicted protein-coding sequences was performed on the basis of BLASTP searches against the NCBI nonredundant database. CDSs were annotated as hypothetical protein-coding genes when they met any of the following four criteria in the top hit of the BLASTP analysis: (1) E-value >1e-8, (2) length coverage <60 % against query sequence (3) sequence identity <30 % or (4) function of the hit was unidentified. The WebMGA server was used to assign the genes to Clusters of Ortholog Groups and Protein family domains [8–11]. The Phobius server was used to predict signal peptides and transmembrane helices [12]. Clustered Regularly Interspaced Short Palindromic Repeat loci were detected using CRISPRfinder [13].

## Genome properties

The basic statistics of the genomes are shown in Table 3. Both genomes contained 46 tRNA genes and one rRNA operon. The genome size of *Sulfurifustis**variabillis* skN76^T^ was approximately 1.4 times larger than that of *Sulfuricaulis limicola* HA5^T^. CRISPR loci were found only in the genome of *Sulfurifustis**variabillis* skN76^T^ (Table 3). The distribution of genes into COGs functional categories is presented in Table 4.

**Table 3:** Genome statistics of *Sulfurifustis variabilis* skN76^T^ and *Sulfuricaulis limicola* HA5^T^

Attribute	*Sulfurifustis variabilis* skN76^T^		*Sulfuricaulis limicola* HA5^T^	
	Value	% of Total	Value	% of Total
Genome size (bp)	3,958,814	100.00	2,864,672	100.00
DNA coding (bp)	3,565,567	90.06	2,567,493	89.63
DNA G + C (bp)	2,670,566	67.46	1,759,557	61.42
DNA scaffolds	1	100.00	1	100.00
Total genes	3913	100.00	2812	100.00
Protein coding genes	3864	98.75	2763	98.26
RNA genes	49	1.25	49	1.74
Pseudo genes	unknown		unknown	
Genes in internal clusters	unknown		unknown	
Genes with function prediction	2930	75.83	2036	73.69
Genes assigned to COGs	2921	75.60	2165	78.36
Genes with Pfam domains	2970	76.86	2208	79.91
Genes with signal peptides	893	23.11	562	20.34
Genes with transmembrane helices	845	21.87	622	22.51
CRISPR repeats	6		0

**Table 4:** Number of genes associated with general COG functional categories

Code	*Sulfurifustis variabilis* skN76^T^		*Sulfuricaulis limicola* HA5^T^		Description
	Value	%age	Value	%age	
J	164	4.24	159	5.75	Translation, ribosomal structure and biogenesis
A	5	0.13	2	0.07	RNA processing and modification
K	191	4.94	130	4.71	Transcription
L	154	3.99	117	4.23	Replication, recombination and repair
B	1	0.03	1	0.04	Chromatin structure and dynamics
D	36	0.93	31	1.12	Cell cycle control, Cell division, chromosome partitioning
V	43	1.11	29	1.05	Defense mechanisms
T	283	7.32	218	7.89	Signal transduction mechanisms
M	265	6.86	210	7.60	Cell wall/membrane biogenesis
N	66	1.71	64	2.32	Cell motility
U	123	3.18	98	3.55	Intracellular trafficking and secretion
O	185	4.79	142	5.14	Posttranslational modification, protein turnover, chaperones
C	265	6.86	192	6.95	Energy production and conversion
G	148	3.83	101	3.66	Carbohydrate transport and metabolism
E	201	5.20	150	5.43	Amino acid transport and metabolism
F	63	1.63	59	2.14	Nucleotide transport and metabolism
H	167	4.32	129	4.67	Coenzyme transport and metabolism
I	90	2.33	65	2.35	Lipid transport and metabolism
P	189	4.89	127	4.60	Inorganic ion transport and metabolism
Q	56	1.45	35	1.27	Secondary metabolites biosynthesis, transport and catabolism
R	394	10.20	247	8.94	General function prediction only
S	346	8.95	230	8.32	Function unknown
-	943	24.40	598	21.64	Not in COGs

## Insights from the genome sequences

In both the genomes of *Sulfurifustis**variabillis* skN76^T^ and *Sulfuricaulis limicola* HA5^T^, genes involved in the sulfur oxidation pathway were identified. The genomes of both strains contain genes of the DSR system related to the oxidation of elemental sulfur to sulfite [14, 15]. They contain a *dsr* gene cluster of identical composition, *dsrABEFHCMKLJOPNR* (SVA_1954-1967, SCL_1274-1261). There are some *dsr* genes outside of the gene cluster, *dsrAB* (SVA_0258-0259, SCL_0256-0257), *dsrS* (SVA_2921, SCL_0781) and *dsrC* (SVA_0281, SVA_0284, SVA_0358, SVA_0917, SVA_0969, SVA_1205, SVA_1793, SVA_1949, SVA_2832, SVA_3655; SCL_0275, SCL_0524, SCL_0785, SCL_1279, SCL_1423, SCL_2646).

As genes encoding proteins involved in oxidation of sulfite to sulfate in the cytoplasm, both genomes contain two copies of the *aprAB* genes encoding an adenosine-5’-phosphosulphate reductase (SVA_2607-2608, SVA_3565-3564; SCL_0600-0601, SCL_2474-2473), along with the *sat* gene encoding a sulfate adenylyltransferase (SVA_3563, SCL_2472) and the *aprM* gene (SVA_2609, SCL_0602). In addition, the genome of *Sulfuricaulis limicola* HA5^T^ contains the *hdrAACB* genes encoding a Hdr (SCL_2523-2520), but that of *Sulfurifustis**valiabilis* skN76^T^ does not. The AprM and Hdr complex are thought to have similar function that interacts with the adenosine-5’-phosphosulphate reductase [16–18]. The genomes also contain the *soeABC* genes (SVA_2734, SVA_2736-2737; SCL_0523-0521), encoding a membrane-bound polysulfide reductase-like iron-sulfur molybdoprotein, which is suspected to be involved in sulfite oxidation in the cytoplasm [19]. Further, the genome of *Sulfurifustis**valiabilis* skN76^T^ contains the *sorAB* genes (SVA_1391-1390) related to the direct oxidation of sulfite to sulfate in the periplasm [20].

For thiosulfate oxidation, both genomes contain the *soxXYZAB* gene cluster (SVA_2999-3003, SCL_2229-2233). Although sulfide oxidation by these bacteria has not been demonstrated, genes related to sulfide oxidation were identified; the *fccAB* (*soxEF*) genes encoding a flavocytochrome *c*/sulfide dehydrogenase (SVA_0067-0066, SVA_3594-3595; SCL_0078-0077) and the *sqr* gene encoding a sulfide:quinone oxidoreductase (SVA_1781, SVA_2675, SVA3205).

**Fig. 3:**
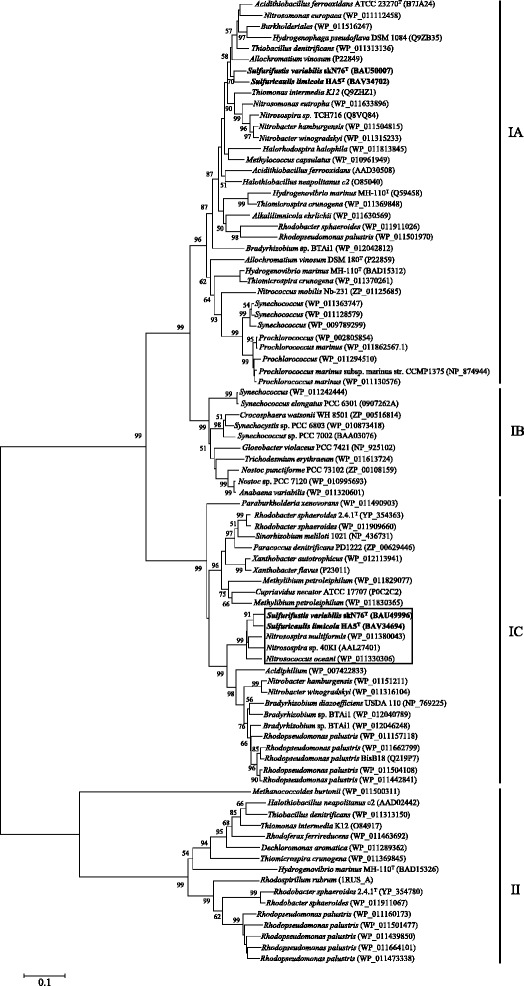
Neighbor-joining tree showing the phylogenetic positions of RuBisCO amino acid sequences coded in the genomes of *Sulfurifustis variabilis* skN76^T^ and *Sulfuricaulis limicola* HA5^T^. The sequences aligned by using CLUSTAL W. This tree was reconstructed using 421 sites with MEGA6 [27]. Percentage values of 1000 bootstrap resamplings are shown at nodes; values below 50 % were not shown. The sequences shown in box have six-amino-acid inserts at the same position

*Sulfurifustis**variabillis* skN76^T^ and *Sulfuricaulis limicola* HA5^T^ are autotrophic bacteria. They both have two copies of the *rbcL* and *rbcS* genes, encoding large and small subunits of ribulose bisphosphate carboxylase/oxygenase (SVA_3460-3459, SVA_3471-3470; SCL_2417-2416, SCL_2425-2424), which is the key enzyme in the Calvin-Benson-Bassham cycle to catalyze inorganic carbon fixation. The two copies of RuBisCO in each genome are phylogenetically distinct, and belong to lineages referred to as green-like form IA and red-like form IC (Fig. 3) [21]. In the form IC RuBisCO coded by *rbcL* gene (SVA_3460, SCL_2417), *Sulfurifustis**variabillis* skN76^T^ and *Sulfuricaulis limicola* HA5^T^ have six-amino-acid inserts at the same position where a similar insert was reported from *Nitrosospira* sp. 40KI [22]. There are two other RuBisCO sequences which have six-amino-acid inserts at the same position, and these sequences with inserts formed a monophyletic cluster in the tree of RuBisCO (Fig. 3). In general, RuBisCO of form IA and IC have different properties which are thought to be advantageous to fix inorganic carbon under different concentrations of carbon dioxide and/or oxygen [21]. Possession of the genes for these two distinct RuBisCO forms may be beneficial to cope with changing environmental conditions, or to thrive in various types of ecosystems.

## Conclusion

This is the first report on complete genome sequences of bacteria belonging to the order *Acidiferrobacterales*. The genome analysis of *Sulfurifustis**variabillis* skN76^T^ and *Sulfuricaulis limicola* HA5^T^ revealed that they have similar sets of genes involved in sulfur oxidation pathways. In the both genomes, redundancies of the genes for sulfur oxidation and inorganic carbon fixation were observed, as represented by multiple copies of *dsrAB*, *aprAB* and *rbcLS*. Such redundancies may provide physiological flexibility to the chemolithotrophic sulfur oxidizers which are fully depending on these functions to obtain energy and carbon source for growth.

## Abbreviations

MiGAP: Microbial Genome Annotation Pipeline
Hdr: Heterodisulfide reductase
DSR: Dissimilatory sulfite reductase

## References

[1] Kojima H, Shinohara A, Fukui M (2015). *Sulfurifustis variabilis* gen. nov., sp. nov., a sulfur oxidizer isolated from a lake, and proposal of *Acidiferrobacteraceae* fam. nov. and *Acidiferrobacterales* ord. nov. Int J Syst Evol Microbiol.

[2] Kojima H, Watanabe T, Fukui M (2016). *Sulfuricaulis limicola* gen. nov., sp. nov., a sulfur oxidizer isolated from a lake. Int J Syst Evol Microbiol.

[3] Hallberg KB, Hedrich S, Johnson DB (2011). *Acidiferrobacter thiooxydans*, gen. nov. sp. nov.; an acidophilic, thermo-tolerant, facultatively anaerobic iron- and sulfur-oxidizer of the family *Ectothiorhodospiraceae*. Extremophiles.

[4] Kelly DP, Wood AP (2000). Reclassification of some species of *Thiobacillus* to the newly designated genera *Acidithiobacillus* gen. nov., *Halothiobacillus* gen. nov. and *Thermithiobacillus* gen. nov. Int J Syst Evol Microbiol.

[5] Williams KP, Kelly DP (2013). Proposal for a new class within the phylum *Proteobacteria*, *Acidithiobacillia* classis nov., with the type order *Acidithiobacillales*, and emended description of the class *Gammaproteobacteria*. Int J Syst Evol Microbiol.

[6] Dyksma S, Bischof K, Fuchs BM, Hoffmann K, Meier D, Meyerdierks A (2016). Ubiquitous *Gammaproteobacteria* dominate dark carbon fixation in coastal sediments. ISME J.

[7] Sugawara H, Ohyama A, Mori H, Kurokawa K. Microbial Genome Annotation Pipeline (MiGAP) for diverse users. The 20th International Conference on Genome Informatics (GIW2009) Poster and Software Demonstrations (Yokohama). 2009;S001-1-2.

[8] Wu S, Zhu Z, Fu L, Niu B, Li W (2011). WebMGA: a customizable web server for fast metagenomic sequence analysis. BMC Genomics.

[9] Altschul SF, Gish W, Miller W, Myers EW, Lipman DJ (1990). Basic local alignment search tool. J Mol Biol.

[10] Eddy SR (1998). Profile hidden Markov models. Bioinformatics.

[11] Finn RD, Mistry J, Tate J, Coggill P, Heger A, Pollington JE (2010). The Pfam protein families database. Nucleic Acids Res.

[12] Kall L, Krogh A, Sonnhammer ELL (2007). Advantages of combined transmembrane topology and signal peptide prediction-the Phobius web server. Nucleic Acids Res.

[13] Grissa I, Vergnaud G, Pourcel C (2007). CRISPRFinder: a web tool to identify clustered regularly interspaced short palindromic repeats. Nucleic Acids Res.

[14] Pott AS, Dahl C (1998). Sirohaem sulfite reductase and other proteins encoded by genes at the *dsr* locus of *Chromatium vinosum* are involved in the oxidation of intracellular sulfur. Microbiology.

[15] Dahl C, Engels S, Pott-Sperling AS, Schulte A, Sander J, Lübbe Y (2005). Novel genes of the *dsr* gene cluster and evidence for close interaction of Dsr proteins during sulfur oxidation in the phototrophic sulfur bacterium *Allochromatium vinosum*. J Bacteriol.

[16] Parey K, Demmer U, Warkentin E, Wynen A, Ermler U, Dahl C (2013). Structural, biochemical and genetic characterization of dissimilatory ATP sulfurylase from *Allochromatium vinosum*. PLoS One.

[17] Pires RH, Lourenço AI, Morais F, Teixeira M, Xavier AV, Saraiva LM (2003). A novel membrane-bound respiratory complex from *Desulfovibrio desulfuricans* ATCC 27774. Biochim Biophys Acta.

[18] Meyer B, Kuever J (2007). Molecular analysis of the distribution and phylogeny of dissimilatory adenosine-5’-phosphosulfate reductase-encoding genes (*aprBA*) among sulfur-oxidizing prokaryotes. Microbiology.

[19] Dahl C, Franz B, Hensen D, Kesselheim A, Zigann R (2013). Sulfite oxidation in the purple sulfur bacterium *Allochromatium vinosum*: identification of SoeABC as a major player and relevance of SoxYZ in the process. Microbiology.

[20] Kappler U, Bennett B, Rethmeier J, Schwarz G, Deutzmann R, McEwan AG (2000). Sulfite:cytochrome *c* oxidoreductase from *Thiobacillus novellus* — purification, characterization and molecular biology of a heterodimeric member of the sulfite oxidase family. J Biol Chem.

[21] Badger MR, Bek EJ (2008). Multiple Rubisco forms in proteobacteria: their functional significance in relation to CO_2_ acquisition by the CBB cycle. J Exp Bot.

[22] Utåker JB, Andersen K, Aakra Å, Moen B, Nes IF (2002). Phylogeny and functional expression of ribulose 1,5-Bisphosphate carboxylase/oxygenase from the autotrophic ammonia-oxidizing bacterium *Nitrosospira* sp. Isolate 40KI. J Bacteriol.

[23] Woese CR, Kandler O, Wheelis ML (1990). Towards a natural system of organisms: Proposal for the domains Archaea, Bacteria, and Eucarya. Proc Natl Acad Sci U S A.

[24] Garrity GM, Bell JA, Lilburn T. Phylum XIV. *Proteobacteria* phyl. nov. In: Garrity GM, Brenner DJ, Krieg NR, Staley JT, editors. Bergey’s Manual of Systematic Bacteriology, Volume 2, Part B. 2nd ed. New York: Springer; 2005. p. 1.

[25] Garrity GM, Bell JA, Lilburn T. Class III. *Gammaproteobacteria* class. nov. In: Garrity GM, Brenner DJ, Krieg NR, Staley JT, editors. Bergey’s Manual of Systematic Bacteriology, Volume 2, Part B. 2nd ed. New York: Springer; 2005. p. 1.

[26] Kojima H, Iwata T, Fukui M (2009). DNA-based analysis of planktonic methanotrophs in a stratified lake. Freshw Biol.

[27] Tamura K, Stecher G, Peterson D, Filipski A, Kumar S (2013). MEGA6: molecular evolutionary genetics analysis version 6.0. Mol Biol Evol.

